# Assessing quality and completeness of human transcriptional regulatory pathways on a genome-wide scale

**DOI:** 10.1186/1745-6150-6-15

**Published:** 2011-02-28

**Authors:** Evgeny Shmelkov, Zuojian Tang, Iannis Aifantis, Alexander Statnikov

**Affiliations:** 1 Department of Pharmacology, New York University School of Medicine, New York, NY, USA; 2 Center for Health Informatics and Bioinformatics, New York University School of Medicine, New York, NY, USA; 3 Howard Hughes Medical Institute and Department of Pathology, New York University School of Medicine, New York, NY, USA; 4 Department of Medicine, New York University School of Medicine, New York, NY, USA

## Abstract

**Background:**

Pathway databases are becoming increasingly important and almost omnipresent in most types of biological and translational research. However, little is known about the quality and completeness of pathways stored in these databases. The present study conducts a comprehensive assessment of transcriptional regulatory pathways in humans for seven well-studied transcription factors: MYC, NOTCH1, BCL6, TP53, AR, STAT1, and RELA. The employed benchmarking methodology first involves integrating genome-wide binding with functional gene expression data to derive direct targets of transcription factors. Then the lists of experimentally obtained direct targets are compared with relevant lists of transcriptional targets from 10 commonly used pathway databases.

**Results:**

The results of this study show that for the majority of pathway databases, the overlap between experimentally obtained target genes and targets reported in transcriptional regulatory pathway databases is surprisingly small and often is not statistically significant. The only exception is MetaCore pathway database which yields statistically significant intersection with experimental results in 84% cases. Additionally, we suggest that the lists of experimentally derived direct targets obtained in this study can be used to reveal new biological insight in transcriptional regulation and suggest novel putative therapeutic targets in cancer.

**Conclusions:**

Our study opens a debate on validity of using many popular pathway databases to obtain transcriptional regulatory targets. We conclude that the choice of pathway databases should be informed by solid scientific evidence and rigorous empirical evaluation.

**Reviewers:**

This article was reviewed by Prof. Wing Hung Wong, Dr. Thiago Motta Venancio (nominated by Dr. L Aravind), and Prof. Geoff J McLachlan.

## Background

Recently the biological pathways have become a common and probably the most popular form of representing biochemical information for hypothesis generation and validation. These maps store wide knowledge of complex molecular interactions and regulations occurring in the living organism in a simple and obvious way, often using intuitive graphical notation. Two major types of biological pathways could be distinguished. Metabolic pathways incorporate complex networks of protein-based interactions and modifications, while signal transduction and transcriptional regulatory pathways are usually considered to provide information on mechanisms of transcription [1].

For the last decade a variety of different public and commercial online pathway databases have been developed [2] and are currently routinely utilized by biomedical researchers and even by the U.S. government regulatory agencies such as FDA [3]. Each of these databases has its own structure, way of data storage and representation, and method for extracting and verifying biological knowledge. Information in the most popular publicly available pathway databases, such as BioCarta, KEGG [4], WikiPathways [5], Cell Signaling Technology pathways is usually either curated by efforts of a particular academic group (e.g., KEGG) or by direct participation of the broader scientific community (e.g., WikiPathways). Popular commercial products, such as MetaCore [6], Ingenuity Pathway Analysis, Pathway Studio and Biobase Knowledge Library (TRANSPATH and TRANSFAC) are often based on literature curation (e.g., Biobase Knowledge Library) and/or complex algorithms for mining biomedical literature (e.g., Pathway Studio).

**Figure 1:**
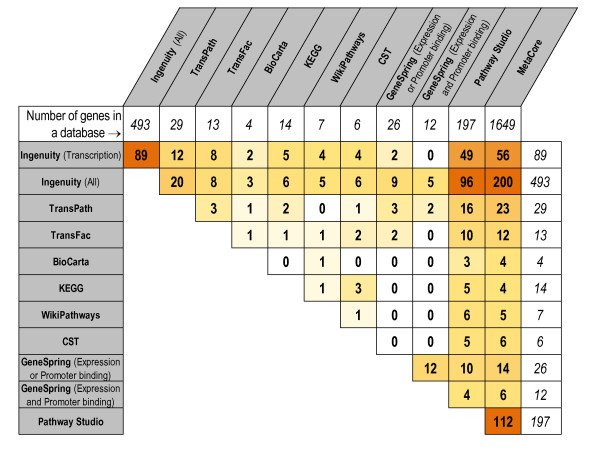
**Number of genes in common between MYC transcriptional targets derived from ten different pathway databases**. Cells are colored according to their values from white (low values) to red (high values).

While there are a lot of data collected on human metabolic processes, the content of signal transduction and transcriptional regulatory pathways varies greatly in quality and completeness [7]. An indicative comparison of MYC transcriptional targets reported in ten different pathway databases reveals that these databases differ greatly from each other (Figure 1). Given that MYC is involved in the transcriptional regulation of approximately 15% of all genes [8], one cannot argue that the majority of pathway databases that contain less than thirty putative transcriptional targets of MYC are even close to complete. More importantly, to date there have been no prior genome-wide evaluation studies (that are based on genome-wide binding and gene expression assays) assessing pathway databases. Thus, biomedical scientists have to make their choice of the database based on interface, prior experience, or marketing presentations. However, it is critically important that this choice is informed by a rigorous evaluation that utilizes genome-wide experimental data. In the current study we perform such an evaluation of ten commonly used pathway databases. Particularly, we assessed the transcriptional regulatory pathways, considered in the current study as the interactions of the type 'transcription factor-transcriptional targets'.

Our study first involves integration of human genome-wide functional microarray or RNA-seq gene expression data with protein-DNA binding data from ChIP-chip, ChIP-seq, or ChIP-PET platforms to find direct transcriptional targets of the seven well known transcription factors: MYC, NOTCH1, BCL6, TP53, AR, STAT1, and RELA. The choice of transcription factors is based on their important role in oncogenesis and availability of binding and expression data in the public domain. Then, the lists of experimentally derived direct targets are used to assess the quality and completeness of 84 transcriptional regulatory pathways from four publicly available (BioCarta, KEGG, WikiPathways and Cell Signaling Technology) and six commercial (MetaCore, Ingenuity Pathway Analysis, BKL TRANSPATH, BKL TRANSFAC, Pathway Studio and GeneSpring Pathways) pathway databases. We measure the overlap between pathways and experimentally obtained target genes and assess statistical significance of this overlap. Additionally, we demonstrate that experimentally derived lists of direct transcriptional targets can be used to reveal new biological insight on transcriptional regulation. We show this by analyzing common direct transcriptional targets of MYC, NOTCH1 and RELA that act in interconnected molecular pathways. Detection of such genes is important as it could reveal novel targets of cancer therapy.

## Methods

### Functional expression data

The functional expression data was obtained from eleven previously published gene expression microarray and RNA-seq studies [9-19], where the transcription factor in question was knocked-down or over-expressed (see Table S1 in Additional File 1). In order to achieve statistical significance of our results, we selected only datasets with at least eight samples in total and three samples per condition.

### Protein-DNA binding data

The transcription factor-DNA genome-wide binding data was derived from seven previously published ChIP-chip, ChIP-seq and ChIP-PET studies summarized in Table S2 in Additional File 1[9,13,14,20-23].

### Generation of the gold-standards

A gold-standard is a list of genes that are directly downstream of a particular transcription factor, and are functionally regulated by it. Generation of gold-standards involved steps that are outlined below.

Functional gene expression data was first used to identify genes that are *downstream (but not necessarily directly) *of a particular transcription factor by application of the Student's t-test with α = 0.05 to 'experiment' (e. g., siRNA) and 'control' samples. Wherever applicable, we used a paired t-test that has larger statistical power to find differentially expressed genes than an unpaired version. Also, if some transcription factor had a well-known role as either activator or repressor for the majority of target genes, we created two gold-standards: one with a one-sided t-test and another one with a two-sided t-test. For example, since it is known that MYC is an activator for most target genes [24], we expect that in siRNA experiments genes downstream of MYC are down-regulated; this can be detected by a one-sided t-test. However, since there are studies that reported role of MYC as a repressor [25,26], we can also expect that genes downstream of MYC can be either up-or down-regulated; this can be detected by a two-sided t-test.

Genome-wide binding data was then employed to identify *direct binding targets *of each transcription factor. Specifically, for each studied transcription factor we obtained the set(s) of genes with detected promoter region-transcription factor binding according to the primary study that generated binding data.

We emphasize that using genome-wide binding data by itself is insufficient to find downstream *functional *targets of a transcription factor, because many binding sites can be non-functional [27]. Therefore, the final step in gold-standard creation required overlapping of the list of *direct binding targets *(from binding data) with the list of *downstream functional targets *(from expression data). Knowledge gained by integration of data from these two sources is believed to provide high confidence that a given transcription factor directly regulates a particular gene [28]. Also, integration of data from two different sources contributes to the reduction of false positives in the resulting gold-standards.

### Pathway databases

In the current study we analyzed twelve pathway-derived sets of direct transcriptional targets for each transcription factor of interest. These gene sets were extracted from the ten pathway databases listed in Table 1 according to the following protocol.

**Table 1:** Pathway databases.

Pathway Database	Access	Primary data source	Vendor/Developer	Web site
Ingenuity Pathway Analyzer (IPA)	Commercial	Expert curation/data mining	Ingenuity Systems, Inc.	http://www.ingenuity.com
BKL TRANSPATH	Commercial	Expert curation	BIOBASE	http://www.biobase-international.com
BKL TRANSFAC	Commercial	Expert curation	BIOBASE	http://www.biobase-international.com
BioCarta	Free	Expert curation/scientific community contribution	BioCarta, LLC	http://www.biocarta.com
KEGG	Free	Expert curation	Kanehisa Laboratories	http://www.genome.jp/kegg
WikiPathways	Free	Expert curation/scientific community contribution	BiGCaT Bioinformatics (University of Maastricht) and Conklin Lab (Gladstone Institutes, UCSF)	http://www.wikipathways.org
Cell Signaling Technology pathways (CST)	Free	Expert curation/scientific community contribution	Cell Signaling Technology, Inc.	http://www.cellsignal.com
GeneSpring	Commercial	Expert curation/data mining	Agilent Technologies, Inc.	http://www.chem.agilent.com
Pathway Studio	Commercial	Data mining	Ariadne Genomics, Inc.	http://www.ariadnegenomics.com
MetaCore	Commercial	Expert curation	GeneGo, Inc.	http://www.genego.com

From each relevant pathway present in BioCarta, KEGG, WikiPathways and Cell Signaling Technology, we manually extracted all stated direct transcriptional targets of each of the seven transcription factors from our list. From the Ingenuity Pathway Analysis database, we extracted two sets of target genes regulated by each transcription factor of interest. One of them (a more conservative) contained all genes with the 'transcription' relation type to a given transcription factor, while another one (a more liberal) incorporated union of the genes with relation types 'transcription', 'expression' and 'protein-DNA interaction'. From each of the BKL TRANSPATH and TRANSFAC databases, we extracted a set of genes that are stated to be regulated by each transcription factor in question (i.e., "Binding Sites/Regulated Genes" and "Regulates expression of (direct or indirect)"). From the GenSpring database, we created two gene sets. The first set (a more conservative) contained intersection of the following two groups of genes: genes regulated by the transcription factor on the expression level and genes that are bound to a given transcription factor. The second set (a more liberal) incorporated a union of above two groups of genes. Finally, from the Pathway Studio and MetaCore pathway databases, we extracted a set of targets that are transcriptionally regulated by each of the transcription factors from our list (in MetaCore we considered a union of genes with 'transcription regulation' and 'co-regulation of transcription' types of relations with given transcription factor).

### Statistical comparison of gene sets

Since we are seeking to compare gene sets from different studies/databases, it is essential to transform genes to standard identifiers. That is why we transformed all gene sets to the HUGO Gene Nomenclature Committee approved gene symbols and names [29].

**Figure 2:**
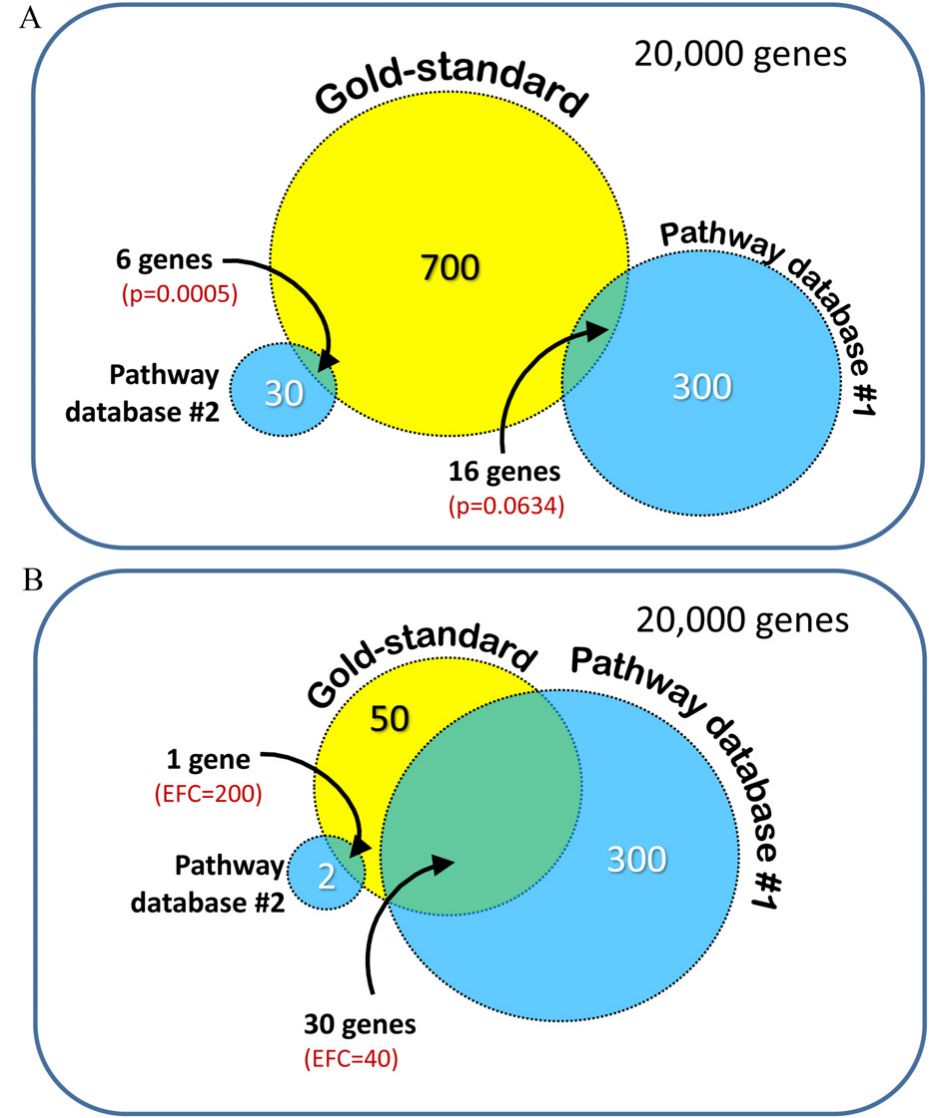
**Illustration of statistical methodology for comparison between a gold-standard and a pathway database**.

In order to assess statistical significance of the overlap between the resulting gene sets, we used the hypergeometric test at 5% α-level with false discovery rate correction for multiple comparisons by the method of Benjamini and Yekutieli [30]. The alternative hypothesis of this test is that two sets of genes (set A from pathway database and set B from experiments) have greater number of genes in common than two randomly selected gene sets with the same number of genes as in sets A and B. For example, consider that for some transcription factor there are 300 direct targets in the pathway database #1 and 700 in the experimentally derived list (gold-standard), and their intersection is 16 genes (Figure 2a). If we select on random from a total of 20,000 genes two sets with 300 and 700 genes each, their overlap would be greater or equal to 16 genes in 6.34% times. Thus, this overlap will not be statistically significant at 5% α-level (p = 0.0634). On the other hand, consider that for the pathway database #2, there are 30 direct targets of that transcription factor, and their intersection with the 700-gene gold-standard is only 6 genes. Even though the size of this intersection is rather small, it is unlikely to randomly select 30 genes (out of 20,000) with an overlap greater or equal to 6 genes with a 700-gene gold-standard (p = 0.0005, see Figure 2a). This overlap is statistically significant at 5% α-level.

Even though the above statistical methodology is based on odds ratios, databases with a very small number of targets may not reach statistical significance regardless of the quality of their data. To address this issue and provide another view on the data of our study, we calculate an enrichment fold change ratio (EFC) for every intersection between a gold-standard and a pathway database. For a given pair of a gold-standard and a pathway database, EFC is equal to the observed number of genes in their intersection, divided by the expected size of intersection under the null hypothesis (plus machine epsilon, to avoid division by zero). Notice however that larger values of EFC may correspond to databases that are highly incomplete and contain only a few relations. For example, consider that for some transcription factor there are 300 direct targets in the pathway database #1 and 50 in the experimentally derived list (gold-standard), and their intersection is 30 genes (Figure 2b). If we select on random from a total of 20,000 genes two sets with 300 and 50 genes each, their expected overlap under the null hypothesis will be equal to 0.75. Thus, the EFC ratio will be equal to 40 (= 30/0.75). On the other hand, consider that for the pathway database #2, there are 2 direct targets of that transcription factor, and their intersection with the 50-gene gold-standard is only 1 gene. Even though the expected overlap under the null hypothesis will be equal to 0.005 and EFC equal to 200 (5 times bigger than for the database #1), the size of this intersection with the gold-standard is 30 times less than for database #1 (Figure 2b).

## Results

### Comparison between pathway databases

First, we assessed all pathway databases listed in Table 1 by a comparison of the extracted transcriptional targets for each of the seven transcription factors. The number of overlapping MYC targets between pathway databases is shown in Figure 1. We also calculated Jaccard index, the normalized measure of similarity between two gene lists (which is the size of intersection between two gene lists divided by the size of their union), for all pairwise comparisons of pathway databases (Figure S1 in Additional File 1). Since the information in the majority of these databases is curated, we would expect to see almost full intersection for every pair of databases, i.e. Jaccard index close to 1. However, our analysis revealed that the transcriptional data differs significantly from one database to another. Indeed, the average Jaccard index over all pairwise comparisons of pathway databases ranges between 0.0180 (AR) and 0.0742 (RELA), depending on a transcription factor. The grand average Jaccard index over all pathway comparisons and transcription factors is 0.0533. This means that only 5.33% of gene targets that belong to either of the two transcriptional regulatory pathways, also belong to both pathways. Furthermore, the transcriptional regulatory information on particular transcription factors was totally absent from some pathway databases.

### Generation of the gold-standards

By integrating functional gene expression and genome-wide binding datasets, we obtained 25 gold-standards for the seven transcription factors considered in our study (Table 2). Each of these gold-standards was generated as described in the Methods section and contains genes that are directly downstream of a particular transcription factor and are functionally regulated by it. A detailed list of all genes in each gold-standard is available from http://www.nyuinformatics.org/downloads/supplements/PathwayAssessment Additionally, as for MYC, NOTCH1, RELA, and BCL6 there was more than one dataset (functional gene expression or genome-wide binding) available, we also generated a list of most confident direct downstream targets of each of these transcription factors by overlapping gold-standards that were obtained with different datasets (Table S3 in Additional File 1). While these lists are expected to contain only the most confident genes which are directly regulated by a transcription factor in question, these lists are likely to be incomplete due to condition-specific transcriptional targets that may not appear in all datasets and also due to statistical considerations, namely the fact that probability of obtaining significant results in several studies declines with the number of studies [31].

**Table 2:** Gold standards for each transcription factor (TF).

TF	Gold Standard ID#	Genes bound by the TF are obtained from	Genes that are downstream of the TF are obtained from	
			
			Study	Analyzed to find genes that are
AR	I			• differentially expressed between DHT treated (for 4 hr.) and control samples
	II	Wang et al. [22]	Wang et al. [18]	• differentially expressed between DHT treated (for 16 hr.) and control samples
	III			• differentially expressed between DHT treated (for 4 hr. and 16 hr.) and control samples

BCL6	I	Basso et al. [9]		• differentially expressed between siRNA treated and control samples
	II	Ci et al [20]	Basso et al. [9]	• differentially expressed between siRNA treated and control samples
	III	Basso et al. [9]		• up-regulated in siRNA treated samples
	IV	Ci et al [20]		• up-regulated in siRNA treated samples

MYC	I		Cappellen et al. [11]	• differentially expressed between siRNA treated and control samples
	II	Margolin et al. [14]		• down-regulated in siRNA treated samples
	III		Bild et al. [10]	• differentially expressed between MYCexpressing and control samples
	IV			• up-regulated in MYC-expressing samples

NOTCH1	I		Margolin et al. [14]	• differentially expressed between GSI treated and control samples
	II			• down-regulated in GSI treated samples
	III	Margolin et al. [14]	Palomero et al. [15]	• differentially expressed between GSI treated and control samples
	IV			• down-regulated in GSI treated samples
	V		Sanda et al. [17]	• differentially expressed between GSI treated and control samples
	VI			• down-regulated in GSI treated samples

RELA	I		Espinosa et al. [19]	• differentially expressed between NBD treated and control samples
	II	Kasowski et al. [13]		• down-regulated in NBD treated samples
	III		Kasowski et al. [13]	• differentially expressed between TNF-α treated and control samples
	IV			• up-regulated in TNF-α treated samples

STAT1	I	Robertson et al. [21]	Pitroda et al. [16]	• differentially expressed between shRNA treated and control samples
	II			• down-regulated in shRNA treated samples

TP53	I	Wei et al. [23]	Chau et al. [12]	• differentially expressed between shRNA and control samples
	II			• down-regulated in shRNA treated samples

### Comparison between gold-standards and transcriptional regulatory pathways

**Figure 3:**
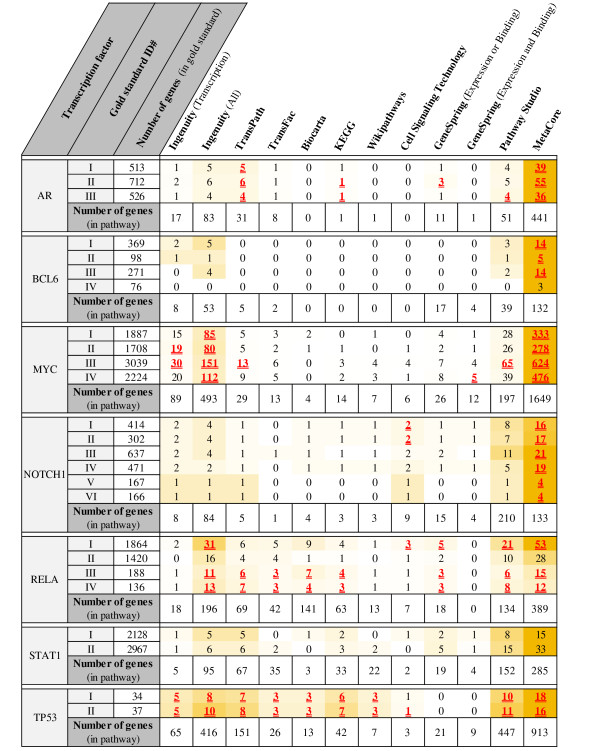
**Comparison between different pathway databases and experimentally derived gold-standards for all considered transcription factors**. Value in a given cell is a number of overlapping genes between a gold-standard and a pathway-derived gene set. Cells are colored according to their values from white (low values) to red (high values). Underlined values in red represent statistically significant intersections.

**Figure 4:**
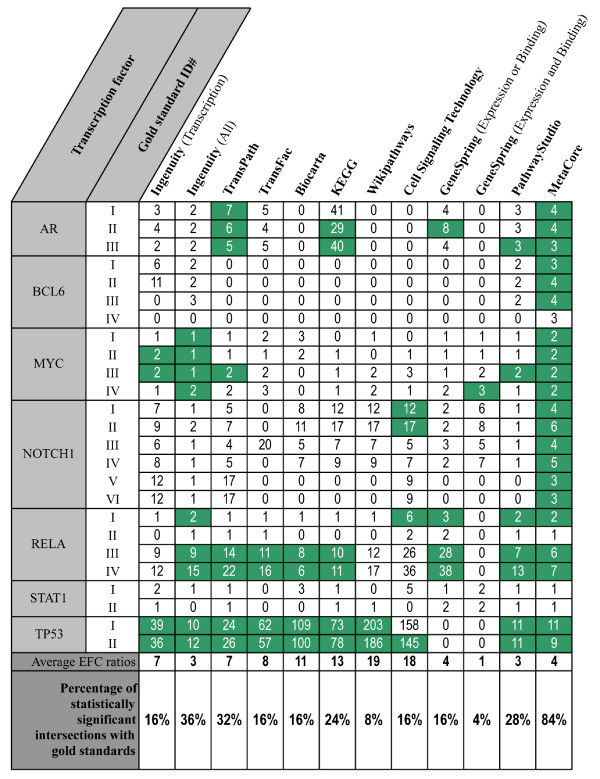
**Summary of the pathway databases assessment**. Green cells represent statistically significant intersections between experimentally derived gold-standards and transcriptional regulatory pathways. White cells denote results that are not statistically significant. Numbers are the enrichment fold change ratios (EFC) calculated for each intersection.

For each transcription factor, we analyzed an overlap between experimentally derived gold-standards of direct transcriptional targets and 12 gene sets from pathway databases as described in the Methods section. The detailed results are shown in Figure 3 and are summarized in Figure 4. Over all gold-standards and transcription factors, the largest overlap with experimentally derived gene targets was obtained by MetaCore (statistically significant overlap with 84% gold-standards). Other pathway databases to a large extent do not have statistically significant overlaps with experimentally obtained target genes: the best of the remaining pathway databases, Ingenuity Pathway Analysis, results in statistically significant overlap with only 36% of gold-standards. Notably, in 82 comparisons (over all 25 gold-standards and 12 gene sets for each transcription factor) the resulting overlap of pathways with gold-standards is empty. In brief, for the majority of pathway databases used in this study, randomly selected gene sets yield the same number of overlapping genes with gold-standards as the pathway databases. On the other hand, the biggest average EFC ratio was obtained by WikiPathways and was equal to 19. Notably, the intersection of WikiPathways data with the gold-standard #I for TP53 was 203 times bigger than the expected one for that overlap (the largest EFC over all assessed data). Furthermore, the EFC of this intersection was approximately 18.5 times bigger than the corresponding EFC of MetaCore. However, the overlap of WikiPathways data with this gold-standard (3 genes) was 6 times smaller than the overlap of MetaCore data with the gold-standard (18 genes).

### Cross-talk of MYC, NOTCH1, RELA transcriptional regulatory pathways

The gold-standards generated in our study contain direct targets of each individual transcription factor. However, these factors only rarely act individually. Indeed, we and others have previously suggested that induction and maintenance of T-cell acute lymphoblastic leukemia (T-ALL), a devastating pediatric blood cancer, depends on the cross talk of three transcription factors, NOTCH1, MYC, and RELA (NF-κB) [15,19,32]. We have thus hypothesized that these factors should share target-genes that could be important for the progression of the disease. Our analysis supported this hypothesis as it identified a large number of genes targeted by two or more factors (Table S4 in Additional File 1). As expected, NOTCH1 and MYC, two transcription factors that have been closely connected in T-ALL share a large number of common targets (> 400). Some of these genes are very intriguing from a biological point of view. For example, two activators of cell cycle entry, CDK4 and CDK6 appear to be induced by both factors. We have previously shown that silencing of CDK4/6 activity is able to suppress T-ALL suggesting that NOTCH1 and MYC activities could converge on these CDK genes to initiate expansion of transformed cells [33]. Interestingly, MYC itself and its interacting partners MYCB and MYCB2 appear to also be targeted by both factors, suggesting an interesting signal amplification mechanism.

RELA and MYC also share a large number (> 550) of common gene targets. This is a novel biological finding with importance for the biology of T-cell leukemia. For example, several essential T-cell regulators, including RUNX1, BCL2L1 (BCL-xL), ID3, ITCH, JAK3 and NOTCH1, appear to be controlled by both transcription factors. Interestingly, NOTCH1 is downstream of both RELA and MYC but at the same time these two factors are targets of oncogenic NOTCH1 [15,32], suggesting once more an intricate auto-amplification loop that could sustain transformation.

## Discussion

At the core of this study was creation of gold-standards of transcriptional regulation in humans that can be compared with target genes reported in transcriptional regulatory pathways. We focused on seven well known transcription factors and obtained gold-standards by integrating genome-wide transcription factor-DNA binding data (from ChIP-chip, ChIP-seq, or ChIP-PET platforms) with functional gene expression microarray and RNA-seq data. The latter data allows to survey changes in the transcriptomes on a genome-wide scale after the inhibition or over-expression of the transcription factor in question. However, change in the expression of a particular gene could be caused either by the direct effect of the removal or introduction of a given transcription factor, as well as by an indirect effect, through the change in expression level of some other gene(s). As mentioned in the Methods section, transcription factor-DNA binding data by itself is similarly insufficient to determine downstream functional targets of a transcription factor due to the occurring nonfunctional and/or non-specific protein binding activity [27]. Thus, it is essential to integrate data from these two sources to obtain an accurate list of gene targets that are directly regulated by a transcription factor [28].

It is worth noting that tested pathway databases typically do not give distinction between cell-lines, experimental conditions, and other details relevant to experimental systems in which data were obtained. These databases in a sense propose a 'universal' list of transcriptional targets. However, it is known that transcriptional regulation in a cell is dynamic and works differently for different systems and stimuli. This accentuates the major limitation of pathway databases and emphasizes importance of deriving a specific list of transcriptional targets for the current experimental system. In this study we followed the latter approach by developing gold-standards for specific well-characterized biological systems and experimental conditions.

However our approach for building gold-standards of direct mechanistic knowledge has several limitations. First of all, these are limitations inherited by the assaying technology. Microarrays cannot reliably detect small changes in gene expression and/or genes expressed on very low levels [34]. Similarly, ChIP-chip transcription factor-DNA binding data is known to have imperfect reproducibility [35]. Second, functional gene expression and binding data used in our work often originated from different studies. Even though we verified that there were comparable biological systems, even minor differences in experimental conditions and phenotypes between studies can challenge integration of binding and functional expression data. Third, the siRNA knock-down is not ideal experiment for proving direct causation for identified binding relations, because it could cause some false positives in our gold-standards. For example, a transcription factor could bind to promoter region of a gene, but regulate expression of that gene indirectly via transregulation of another transcription factor. Forth, some number of false negatives in our approach could arise due to the compensatory mechanism in the cell. Finally, our approach can yield only gold-standards with direct downstream targets of the transcription factor, and therefore information about upstream regulation cannot be obtained by this method. Likewise, it does not allow capturing interactions between genes that are not transcription factors. Notwithstanding the above challenges, this method is currently state-of-the-art and is believed to provide with high confidence direct regulatory interactions of the transcription factors on genome-wide scale [28].

In addition to assessment of pathway databases, these gold-standards can be used in order to get new biological insights in the field of transcriptional regulation. Specifically, our results suggest that multiple transcription factors can co-operate and control both physiological differentiation and malignant transformation, as demonstrated utilizing combinatorial gene-profiling for NOTCH1, MYC and RELA targets. These studies might lead us to multi-pathway gene expression "signatures" essential for the prediction of genes that could be targeted in cancer treatments. In agreement with this hypothesis, several of the genes identified in our analysis have been suggested to be putative therapeutic targets in leukemia, with either preclinical or clinical trials underway (CDK4, CDK6, GSK3b, MYC, LCK, NFkB2, BCL2L1, NOTCH1) [36].

## Conclusions

The comparison of the pathway databases, the main goal of this study, first of all revealed that human transcriptional regulatory pathways often do not agree with each other and contain different target genes. More importantly, with the exception of MetaCore, the majority of sets of target genes specified in the transcriptional regulatory pathways were found to be incomplete and/or inaccurate when compared with experimentally derived gold-standards. Despite of the fact that in the present study we assessed the transcriptional pathways of only seven transcription factors (due to limited availability of high-quality genome-wide binding and expression data in the public domain), we anticipate that our results would generalize to other transcription factors and pathways.

Given widespread use of pathways for hypothesis generation, the conclusions of our study have significant implications for biomedical research in general and discovery of new drugs and treatments. In order to obtain a more accurate research hypothesis, the choice of pathway databases has to be informed by solid scientific evidence and rigorous empirical evaluations such as ours. We thus aim to continue comprehensive benchmarking of biological pathways to facilitate evidence-based pathway selection of biomedical researchers. At the same time we propose to developers of pathways databases to take advantage of recently available genome-wide binding and functional expression data to refine transcriptional regulatory pathways.

## Competing interests

The authors declare that they have no competing interests.

## Authors' contributions

Conceived and designed the experiments: ES, AS. Performed the experiments: ES, AS, ZT. Analyzed the results of experiments: ES, AS, ZT, IA. Wrote the paper: ES, AS, ZT, IA. All authors read and approved the final manuscript.

## Reviewers' comments

Reviewer #1: Prof. Wing Hung Wong

Department of Statistics, Stanford University, Stanford, CA, USA

### Reviewer's comments

The purpose of this paper is to assess the quality and completeness of information in several popular pathway databases on the targets of transcription factors. To do this, the authors extracted this information for seven transcription factors from 10 pathway databases, and compared it to "gold standard" target lists identified from expression profiling and ChIP-seq experiments. The "gold standard" criterion is a relatively stringent one that requires not only ChIP-seq support for interaction between the regulator and the regulatory regions of the target gene, but also significant changes in target gene expression upon knockdown or overexpression of the regulator.

The main finding is that there is a very low degree of agreement among the target lists extracted from the different databases, and only one of the database (MetaCore) can provide a statistically significant overlap of targets when compared to the gold standard lists.

This is a very timely research. Many investigators are now incorporating information from the pathway databases in their data analysis. The results of this paper serve to warn us that the pathway databases are far from complete and may yield misleading results.

One unsatisfactory aspect of the statistical analysis presented in this paper is the over-reliance of test of significance as opposed to quantification of the degree of overlap by appropriate indexes such as odds ratio or enrichment fold-changes. For example, it may be that TransFac and KEGG annotations are highly reliable but they cannot reach statistical significance because they contain very few relations. From this consideration it is not surprising that MetaCore, which contains a large number of relations, appears to do so much better than the other databases. I hope the authors can address this issue carefully in their revision.

#### Authors' response

We agree with the reviewer that the statistical methodology has to incorporate odds ratios in order to account for differences in sizes of pathway databases. As a matter of fact, our statistical methodology is based on odds ratios and thus is appropriate for the study. We admit that this was not clearly explained in the original paper, and we have clarified this issue in the revised manuscript. Specifically, we made changes to the subsection "Statistical comparison of gene sets" and added Figure 2.

### Reviewer's comments

The authors have not addressed my previous comment:

"One unsatisfactory aspect of the statistical analysis presented in this paper is the over-reliance of test of significance as opposed to quantification of the degree of overlap by appropriate indexes such as odds ratio or enrichment fold-changes. For example, it may be that TransFac and KEGG annotations are highly reliable but they cannot reach statistical significance because they contain very few relations. From this consideration it is not surprising that MetaCore, which contains a large number of relations, appears to do so much better than the other databases. I hope the authors can address this issue carefully in their revision."

In their response they stated that odds ratio is used in their statistics. However this is used only to compute the hypergeometric p-value. As pointed out in my original review, such p-values are very sensitive to the sample size so the databases with a small smaller number of annotations will not be able to reach the threshold of statistical significance. Thus the comparison between the databases, such as the conclusion that MetaCore is more reliable than the other ones, will be biased by this effect. At the very least, the authors should provide in their various tables, the enrichment fold change which is the observed number in the intersection, divided by the expected number (given fixed marginal counts for each factor) under the null hypothesis.

#### Authors' response

We agree with the reviewer that databases with a very small number of reported targets might not reach the threshold of statistical significance, even though their data could be highly reliable. We improved the manuscript accordingly and provided an alternative enrichment fold change metric suggested by the reviewer. Please see the last paragraph of the Methods section, Figure 2b, and Figure 4.

Reviewer #2: Dr. Thiago Motta Venancio (nominated by Dr. L Aravind)

Computational Biology Branch, NCBI, NLM, NIH, Bethesda, MD, USA

### Reviewer's comments

In this paper Shmelkov et al. analyze the targets of 7 important human transcription factors. They found that the data obtained from different databases and direct experimental works are not well correlated, which could raise important concerns given the widespread use of such data repositories by the scientific community. I have some critical points that should be addressed in order to improve clarity and coherence of the manuscript.

1) The authors extracted data from different databases and datasets. How did they handle the problem of different identifier to name genes (e.g. Entrez GeneID, Ensembl)? In addition, what is the impact of different genome versions between databases/datasets in this analysis? These problems could easily give the low overlap found in the manuscript.

2) Many (if not all) of the databases used in the present study import data from the literature. Are the datasets used present in any of the datasets? If yes, this could be an artificial explanation for the MetaCore large overlap would be expected. If not, why are they not there and why the method used by the authors is better than the ones used by the database teams to identify the real transcription factor targets?

3) I think the T-test and the significance level used to define gold standards are inappropriate for genome-scale analysis, due to multiple testing. Did the authors do any statistical analysis to circumvent such problems (e.g. p-value correction)?

4) Some databases have very low numbers of genes. Wouldn't this result in non significant results regardless of the size of the overlap?

5) The human genome has more than 1500 transcription factors. Due to data availability, the authors assessed 7 of them in this study. In my opinion this far from being "genome wide" in the transcription factor landscape.

Minor point: In the abstract the authors say: "and targets reported in transcriptional regulatory pathways is surprisingly small". Are you referring to transcriptional regulatory pathways databases?

#### Authors' response

The point-by-point response follows:

1) As described in the revised manuscript, we have transformed all gene identifiers from different studies/databases to the HUGO Gene Nomenclature Committee approved gene symbols and names (see the first sentence in the subsection "Statistical comparison of gene sets"). We also ensured that we used the same version of the reference genome as used in the original studies when we generated gene lists (e.g., from ChIP-Seq studies).

2) We agree with the reviewer that the above questions can facilitate understanding the results of our study. Indeed, if some database used all recent available data in the literature and analyzed it using appropriate statistical and bioinformatics methodologies, it would have a significant overlap with our gold-standards. As we have found, this was not the case for most tested databases. Knowing how databases obtained their knowledge bases would potentially allow to delve deeper and address the questions posed by the reviewer. However, these questions are outside the scope of our manuscript; its main purpose was to evaluate pathway databases and identify ones that mostly correlate with the experimental data. Finally, it is also worthwhile to mention that most of used databases use proprietary algorithms and do not disclose how they extract underlying knowledge.

3) In this study we build gold-standards by intersecting the list of binding targets (from ChIP-seq/ChIP-chip/Chip-PET data) with the list of differentially expressed downstream targets (from gene expression data). Indeed, we used t-test for analysis of gene expression data with the 5% alpha level. In general this would entail 5% of false positives which can be a fairly large number. Notice however that our criterion for a gene to participate in the gold-standard is not only that it has p-value <0.05 according to the t-test in the gene expression data, but also that it is bound by the transcription factor of interest, as determined from an independent analysis of the ChIP-seq/ChIP-chip/Chip-PET data. Thus, we reduce the number of false positives in the resulting gold-standards. In order to further clarify this in the revised manuscript, we added a sentence in the last paragraph of the subsection "Generation of the gold-standards" of "Methods".

4) We have clarified this issue in the revised manuscript in the subsection "Statistical comparison of gene sets". In summary, the statistical test is based on odds ratios and thus accounts for different number of genes in pathway databases.

5) We used the term "genome-wide" to denote that the gold-standard for each transcription factor was obtained on a genome-wide scale, i.e. it was based on chromatin immunoprecipitation coupled with the entire genome sequencing or microarray gene expression analysis versus focusing on a selected subset of genes. We have improved the third paragraph of the Background section in the revised manuscript to clarify this issue.

Reviewer's Minor point: We agree with the reviewer and have modified the abstract accordingly.

Reviewer #3: Prof. Geoff J McLachlan

Department of Mathematics, The University of Queensland, Brisbane, Australia

This reviewer provided no comments for publication.

## Supplementary Material

Additional file 1 **Supplementary Information**. **Table S1: **Functional gene expression data. **Table S2: **Transcription factor-DNA binding data. **Table S3: **Most confident direct transcriptional targets of each of the four transcription factors. These targets were obtained by overlapping several gold-standards obtained with different datasets for the same transcription factor. **Table S4: **Genes directly regulated by two or more of the three transcription factors: MYC, NOTCH1, and RELA. **Figure S1: **Comparison of gene sets of transcriptional targets derived from ten different pathway databases by Jaccard index. In case, where Jaccard index of an overlap could not be determined due to comparison of two empty gene lists, we assigned value 0. Cells are colored according to the Jaccard index, from white (Jaccard index equal to 0) to dark-orange (Jaccard index equal to 1). Each sub-figure gives results for a different transcription factor: **(a) **AR, **(b) **BCL6, **(c) **MYC, **(d) **NOTCH1, **(e) **RELA, **(f) **STAT1, **(g) **TP53. Click here for file
