# Supplementary material for: Assessing quality and completeness of human transcriptional regulatory pathways on a genome-wide scale
Source: Biol Direct. 2011 Feb 28;6:15. doi: 10.1186/1745-6150-6-15 (PMC3055855; doi:10.1186/1745-6150-6-15)
Supplement: Additional file 1 — Supplementary Information. Table S1: Functional gene expression data. Table S2: Transcription factor-DNA binding data. Table S3: Most confident direct transcriptional targets of each of the four transcription factors. These targets were obtained by overlapping several gold-standards obtained with different datasets for the same transcription factor. Table S4: Genes directly regulated by two or more of the three transcription factors: MYC, NOTCH1, and RELA. Figure S1: Comparison of gene sets of transcriptional targets derived from ten different pathway databases by Jaccard index. In case, where Jaccard index of an overlap could not be determined due to comparison of two empty gene lists, we assigned value 0. Cells are colored according to the Jaccard index, from white (Jaccard index equal to 0) to dark-orange (Jaccard index equal to 1). Each sub-figure gives results for a different transcription factor: (a) AR, (b) BCL6, (c) MYC, (d) NOTCH1, (e) RELA, (f) STAT1, (g) TP53. [file 1745-6150-6-15-S1.PDF]

Supplementary Information

Table S1: Functional gene expression data.

Transcription Factor		Experimental Data				
Name	Primary type of activity	Reference	Sample size	Samples per group	Assay platform	Experimental design details
AR	N/A	Wang et al., Molecular Cell, 2007	9	6 'experiment', 3 'control'	Affymetrix Human Genome U133 Plus 2.0 Array	LNCaP cell-line, treated with DHT for 4 hrs (DHT), and 16 hrs (DHT), or untreated control
BCL6	Repressor	Basso et al., Blood, 2009	8	4 'experiment', 4 'control'	Affymetrix Human Genome U95 Version 2 Array	Burkitt lymphoma cell lines, siRNA targeting BCL6 or control siRNA
MYC	Activator	Cappellen et al., EMBO Reports, 2007	20	10 'experiment', 10 'control'	Affymetrix Human Genome U133 Plus 2.0 Array	4 Human Cancer cell-lines, siRNA targeting LacZ (Control) and c-MYC (c-MYC KD) transfection
		Bild et al., Nature, 2006	20	10 'experiment', 10 'control'	Affymetrix Human Genome U133 Plus 2.0 Array	Primary mammary epithelial cell cultures expressing oncogene or GFP control
NOTCH1	Activator	Margolin et al., PNAS, 2009	20	10 'experiment', 10 'control'	Affymetrix Human Genome U133A 2.0 Array	10 T-ALL cell lines, apply GSI or DMSO (Control)
		Palomero et al., PNAS, 2006	40	20 'experiment', 20 'control'	Affymetrix Human Genome U133 Plus 2.0 Array	10 T-ALL cell lines x 2 replicates each, apply GSI or DMSO (Control)
		Sanda et al., Blood, 2009	8	4 'experiment', 4 'control'	Rosetta/Merck Human 44k 1.1 microarray	4 T-ALL cell lines, apply GSI or DMSO (Control)
RelA	Activator	Espinosa et al., Cancer Cell, 2010	20	10 'experiment', 10 'control'	Affymetrix Human Genome U133 Plus 2.0 Array	5 T-ALL cell lines x 2 replicates each, apply NBD or DMSO (Control)
		Kasowski et al., Science, 2010	20	10 'experiment', 10 'control'	Illumina Genome Analyzer II (RNA-seq)	10 lymphoblastoid cell lines, TNF treated or untreated control
STAT1	Activator	Pitroda et al., BMC Medicine, 2009	8	4 'experiment', 4 'control'	Affymetrix Human GeneChip(R) Gene 1.0 ST Array	Human squamous cell carcinoma cell line, irradiated or untreated control, x 2 replicates each, transfected with shRNA targeting STAT1 or control vector
TP53	Activator	Chau et al., Cancer Research, 2009	10	5 'experiment', 5 'control'	Rosetta/Merck Human 3.0 A1	5 cancer cell lines, transfected with shRNA targeting TP53 or control

Note: Complete references are provided in the main manuscript.

Table S2: Transcription factor-DNA binding data.

Transcription factor name	Reference	Experiment type	Assay platform	Experimental design details
AR	Wang et al., Cell, 2009	ChIP-on-chip	Affymetrix Human Tiling 2.0R Array Set	LNCaP cell-line, treated with DHT
BCL6	Basso et al., Blood, 2009	ChIP-on-chip	Agilent Technologies Human Promoter ChIP-on-chip Microarray Set	Germinal center B cells
	Ci et al., Blood, 2009	ChIP-on-chip	NibleGen promoter arrays (human genome, version 35, May 2004)	Germinal center B cells and DLBCL cell lines OCI-Ly1, OCI-Ly7
MYC	Margolin et al., PNAS, 2009	ChIP-on-chip	Agilent Human Proximal Promoter Microarray	T-ALL cell line HPB-ALL
NOTCH1	Margolin et al., PNAS, 2009	ChIP-on-chip	Agilent Human Proximal Promoter Microarray	T-ALL cell lines CUTLL1 and HPB-ALL
RelA	Kasowski et al., Science, 2010	ChIP-sequencing	Illumina Genome Analyzer II	10 lymphoblastoid cell lines, treated with TNF
STAT1	Robertson et al., Nature Methods, 2007	ChIP-sequencing	Illumina 1G Analyzer	HeLa S3 cell-line; interferon-γ (IFN-γ)–stimulated and unstimulated
TP53	Wei et al., Cell, 2006	ChIP-PET	-	HCT116 colorectal cancer cell-line treated with 5-fluorouracil

Note: Complete references are provided in the main manuscript.

Table S3: Most confident direct transcriptional targets of each of the four transcription factors. These targets were obtained by overlapping several gold-standards obtained with different datasets for the same transcription factor.

Transcription factor	Number of target genes	Gene symbols of target genes
MYC	636	AASDHPPT, ABCB7, ABCB9, ABCC4, ABCC5, ABCD2, ABCF1, ABLIM2, ACRV1, ACSL5, ACTR5, ACVR1C, ADAM17, ADAT1, ADCK1, ADSL, AFF3, AGA, AGGF1, AHI1, AK2, ALDH1B1, ALG3, ANAPC1, ANAPC5, ANK1, ANKH, ANKRD11, ANKRD13C, ANKRD40, ANP32B, AP3S2, APC, APOL2, APTX, ARHGDIA, ARIH2, ARL6IP4, ARMC6, ARPC5L, ARRDC2, ATAD3A, ATE1, ATF4, ATF5, ATP2A3, ATP5F1, ATP5G2, ATP5J2, AUP1, B3GALT6, BAG1, BAG5, BANP, BAT2, BAT5, BCAT1, BCL11B, BCR, BET1, BET1L, BICD1, BMP6, BRD2, BUB3, BYSL, C11orf31, C14orf101, C14orf106, C14orf126, C14orf133, C16orf35, C18orf1, C18orf55, C18orf8, C19orf2, C19orf28, C1QBP, C1orf107, C1orf25, C1orf57, C1orf71, C21orf57, C21orf91, C22orf9, C2orf18, C6orf125, C6orf150, C6orf48, C6orf57, C6orf89, C7orf26, C9orf64, CALR, CAMK1D, CANX, CAPN7, CARHSP1, CBX6, CCDC21, CCNH, CCT7, CCT8, CD2BP2, CD300A, CDADC1, CDC42EP3, CDC7, CDKL3, CDYL, CETN3, CFDP1, CHD1L, CHERP, CHST10, CIAPIN1, CIRBP, CLCC1, CLN6, CLPP, CLTA, CNKSR3, CNNM2, CNOT2, COMMD5, COPS8, COX10, CREB3L4, CRTAP, CSNK2A1, CSNK2B, CST3, CSTF2T, CTSC, CTSS, CUGBP1, CUGBP2, CUL5, CYCS, DAD1, DAZAP2, DCLRE1A, DDHD1, DDX11, DDX18, DDX19A, DDX50, DDX52, DEAF1, DGKE, DHODH, DHX15, DHX33, DHX37, DHX57, DIAPH2, DNAH11, DNAJA3, DNAJB9, DNAJC1, DPH5, DPM2, DPP3, DPYSL5, DTNA, DUS2L, DUS4L, DUSP19, DUT, EGR1, EIF4A1, EIF4B, ELAC1, ELAC2, ELMOD2, ENY2, EPC1, EPM2A, ESD, ETS2, EVL, EXOSC2, EXOSC8, EZH1, FAM60A, FANCF, FAU, FBXL6, FBXW2, FEM1A, FEN1, FIP1L1, FLAD1, FLCN, FMNL3, FTSJ3, FXC1, FXR2, G6PC3, GAD2, GART, GDF11, GEMIN5, GFM1, GFM2, GHITM, GLT25D1, GLTSCR2, GNA12, GNL3L, GOPC, GPHN, GPR107, GRB2, GRINL1A, GSPT1, GTF3A, GTF3C4, GTF3C5, GTPBP4, H2AFX, HADHA, HCFC1, HEATR1, HELLS, HEMGN, HES1, HES4, HIBADH, HINT1, HIRA, HLCS, HM13, HMGB1, HMGN4, HNMT, HRSP12, HS2ST1, HSPA8, HSPBAP1, HSPD1, IFI16, IL15RA, ILVBL, IMMP2L, INHBE, INPP5D, ITGB1, IVD, JMJD4, JTB, KATNB1, KCTD12, KCTD13, KIAA0090, KIAA0664, KIAA1826, KIDINS220, KLF1, KLF16, KLHL9, KPNA5, KPNA6, KPNB1, KPTN, KRAS, LAPTM4B, LDHB, LDLR, LEF1, LMBR1, LRPPRC, LYPLA2, MANEA, MAP2K2, MAP2K5, MAP3K7IP1, MAPRE2, MARCH8, MARS2, MCCC2, MCM3, MCM7, MCTS1, MDM2, MIF, MINA, MKI67IP, MLF1IP, MLF2, MRPL10, MRPL17, MRPL24, MRPL27, MRPL30, MRPL35, MRPL39, MRPL4, MRPL50, MRPL51, MRPL52, MRPS21, MRPS27, MSH3, MSH6, MSTO1, MTDH, MTO1, MTRF1, MTUS1, MYO18B, NADK, NANS, NAP1L1, NAP1L4, NASP, NAT5, NCBP1, NCDN, NCKIPSD, NDUFA11, NDUFAB1, NDUFC1, NDUFS5, NDUFS8, NECAP1, NEK4, NFIB, NFYA, NGLY1, NKD1, NLN, NOL6, NOTCH1, NPAT, NSD1, NSUN3, NSUN4, NUCKS1, NUDCD1, NUFIP1, NUP188, NUP37, NUP43, NUP88, OCIAD1, OSBPL11, OSGEPL1, OSTM1, OXR1, P2RX5, PA2G4, PABPC4, PARN, PCGF5, PCM1, PDAP1, PDCD5, PDCL3, PDE8A, PDIA4, PDIK1L, PELO, PER2, PEX3, PEX5, PGAM1, PHF15, PHF20, PHPT1, PIK3C3, PIK3R2, PKLR, PLA2G12A, PLA2G6, PLAGL1, PLK4, PLXNC1, PNN, POFUT1, POLE4, POLH, POLR2C, POLR2E, POLR3E, POLR3G, POU6F1, PPIA, PPID, PPIG, PPIL6, PPP6C, PRCP, PRDM15, PRMT7, PRPF18, PRPS2, PSAT1, PSEN2, PSMA4, PSMC5, PSMD7, PSMD9, PTBP1, PTMA, PTPN2, PTPRA, PUS3, PVR, QKI, QTRTD1, RAB12, RAB14, RAD51C, RAPGEF6, RBBP4, RBBP6, RBM17, RCC1, RCL1, RDH10, RERE, REXO1, RFWD2, RGS12, RHOBTB3, RHOF, RIC8B, RIF1, RIN3, RING1, RNF125, RNF135, RNF168, RNF26, RNF7, RNPS1, RPL10, RPL10A, RPL13A, RPL15, RPL18, RPL22, RPL24, RPL27, RPL31, RPL4, RPL7L1, RPP14, RPS15A, RPS2, RPS27A, RPS6, RSL1D1, RUFY2, RUNX2, RUNX3, RUVBL2, RYK, SAP18, SARS, SBF2, SCAND2, SCLY, SDCCAG3, SDCCAG8, SDHD, SEH1L, SENP5, SEPHS1, SEPHS2, SF1, SF3A2, SFRS1, SFRS15, SFRS7, SFXN4, SHB, SIL1, SIP1, SKIV2L2, SKP2, SLC25A11, SLC27A1, SLC29A1, SLC2A13, SLC30A7, SLC35D1, SLC38A5, SLC39A3, SLC39A8, SLC5A6, SMAD4, SMARCA2, SMARCE1, SMCR8,

		SMEK2, SMYD2, SNAPC4, SNAPC5, SNRPA1, SNRPE, SNRPF, SNX5, SORD, SOX4, SPATA5L1, SPTLC1, SRR, SSBP1, SSBP4, SSR1, SSRP1, ST3GAL3, ST7L, STIP1, STK4, STOML1, STRAP, STX6, SUPV3L1, TAF1, TAF10, TAF11, TAF12, TAF6L, TAGAP, TBC1D15, TBC1D7, TBL1X, TBPL1, TCEB1, TCERG1, TCF4, TCOF1, TCP1, TDG, TFDP2, TFIP11, TFRC, TGIF2, TH1L, THADA, THOC1, THOP1, THUMPD1, TIGD4, TIMM8A, TIMM9, TIPRL, TIRAP, TK2, TKT, TM7SF3, TMED9, TMEM18, TMEM33, TMEM39A, TMEM41A, TMEM67, TMEM68, TNPO1, TP53RK, TPP2, TPR, TRIB3, TRNT1, TROVE2, TSGA10, TSPAN12, TSTA3, TTBK2, TTC4, TTLL5, TUBGCP2, TUBGCP6, TUSC2, U2AF2, UBASH3A, UBE2D4, UBE2G1, UBE2G2, UBE2M, UBE3C, UBIAD1, UBL5, UBOX5, UCHL5, UCRC, UMPS, UPF2, USP13, USP2, UST, UTP14A, UXS1, UXT, WDR18, WDR33, WDR36, WDR62, WDR75, WHSC1L1, WT1, WWOX, XPNPEP1, XRCC5, YIPF6, YY1, ZBP1, ZBTB40, ZC3H3, ZC3H8, ZCCHC7, ZDHHC17, ZFY, ZNF215, ZNF23, ZNF234, ZNF248, ZNF259, ZNF330, ZNF37A, ZNF397, ZNF41, ZNF44, ZNF485, ZNF529, ZNF561, ZNF576, ZNF614, ZNF628, ZNF75A, ZNRD1, ZRANB3, ZZZ3
BCL6	39	ARL3, B2M, BPGM, BRPF1, C17orf91, CALM3, CDC14A, CLIC1, CLOCK, CTNND1, DDB2, DOHH, EDEM1, EEF1A1, EIF5B, ERCC2, ETV6, FAIM3, GNB2, GRK6, GSK3A, HIST1H2AE, HIST1H3I, HIST1H4J, HIST1H4K, HIST2H2AA3, ID3, LAMC1, LDLR, NME1, PPP1R11, PRMT1, RPL15, RPL19, RPS18, SEC61G, SNRPC, SOCS2, TLR1
NOTCH1	61	ABCF2, ABT1, AHCY, ALG8, APEX1, ATP5G1, BYSL, C1orf109, C8orf33, CCT3, CDK4, CHCHD3, CLN6, COG8, COX5B, DDX56, EIF2S1, EIF5A, EIF5B, EXOSC5, GTF2F2, GTPBP4, ILF3, IMP4, IPO4, KHSRP, KPNA3, LARS, LAS1L, MRPL24, MRPL4, MRPS12, MRPS28, MRPS33, MYC, NDUFS7, NME1, NUTF2, NXT1, PA2G4, PAICS, PAK1IP1, PDCL3, PFAS, PMAIP1, POLR2I, POLR3E, PPIH, PPRC1, PSMB2, PSMB5, PSPH, RPS6KB1, SHQ1, SNAPC5, SSSCA1, TBRG4, TCP1, TIMM9, UCHL5, UTP14A
RELA	35	C12orf52, C14orf118, C16orf58, C3orf31, C3orf42, CD40, CHMP4B, DPH1, EARS2, ETV6, FAM105B, GALE, GLYCTK, GRIPAP1, GSTP1, HIST1H2AG, IGHMBP2, IKBKE, KCTD11, KIAA1967, LRPAP1, MTERFD2, ODF2, PRPF31, PRPF40B, PRR3, PRRT3, SYTL3, TOMM22, TXN2, USE1, WDR4, ZMIZ2, ZNF397, ZNF79

Note: In order to obtain reliable lists of genes for each transcription factor, we overlapped only those gold standards, which were obtained by applying a one-sided t-test for the functional gene expression analysis.

Table S4: Genes directly regulated by two or more of the three transcription factors: MYC, NOTCH1, and RELA.

Transcription factors	Number of target genes	Gene symbols of target genes
MYC and NOTCH1	438	AAK1, AAMP, AARS, AATF, ABCF1, ABCF2, ABT1, ACTR6, ADPGK, AGPAT5, AHCY, AKT1S1, ALG14, ALG8, AMD1, ANAPC13, ANKRD16, AP2S1, AP4B1, APEX1, APTX, ARMC6, ATF4, ATF5, ATM, ATP5B, ATP5G1, ATP5G2, ATP6V1F, BAT1, BAT2D1, BAZ1B, BET1, BRMS1L, BTF3, BYSL, C11orf24, C12orf11, C15orf24, C18orf22, C19orf6, C1orf109, C1orf144, C22orf9, C7orf11, C8orf33, C8orf41, C9orf41, C9orf72, CA11, CACYBP, CBLL1, CCDC12, CCNA2, CCNH, CCT3, CCT8, CDC27, CDK4, CDK6, CHCHD3, CHCHD4, CHERP, CHORDC1, CLN6, CLTA, COG8, COPE, COPS3, COPS7A, COQ4, CORO7, COX5B, CREB1, CSNK1A1, CSNK2A1, CSTF3, CXXC1, CYCS, DAXX, DCLRE1C, DDX18, DDX19A, DDX21, DDX27, DDX50, DDX51, DDX56, DHPS, DHX34, DHX9, DNAJA1, DNAJC19, DULLARD, DUS3L, EAF2, EFTUD2, EIF2B5, EIF2S1, EIF4B, EIF4E, EIF5A, EIF5B, ELAC2, ELP3, ENSA, ERCC4, ESRRA, EXOSC5, EXOSC9, FAM60A, FBXL14, FDXR, FEM1A, FEN1, FIP1L1, FOS, FOXP3, FTSJ3, FUS, FXR1, FXR2, GGA1, GNB2L1, GOPC, GORASP2, GPHN, GRPEL2, GSK3B, GTF2F1, GTF3C2, GTPBP4, HDHD1A, HELLS, HES1, HGS, HINT1, HM13, HN1, HNRPDL, HSPA4, HSPA8, HSPD1, HSPH1, HTATSF1, ILF3, IMP3, IMP4, INO80, IPO11, IPO4, KHSRP, KIAA0406, KIAA1919, KIF9, KPNB1, LAP3, LARS, LAS1L, LOC93622, LRRC41, LSM4, LSM8, LYAR, MAD2L1, MAK10, MAP2K7, MAP3K7, MAPK7, MAPKAP1, MBD1, MCART6, MCM10, MCM5, MCM6, MCTS1, MDM4, MED28, MPDU1, MRPL1, MRPL10, MRPL17, MRPL2, MRPL22, MRPL24, MRPL34, MRPL36, MRPL4, MRPL50, MRPS12, MRPS28, MRPS33, MRPS9, MSH5, MTDH, MTHFD1, MTHFR, MTRF1L, MYC, MYCBP, MYCBP2, Magmas, NARS, NCLN, NDUFA11, NDUFA12, NDUFA6, NDUFA7, NDUFB11, NDUFB8, NDUFS7, NDUFS8, NFE2L1, NFX1, NKTR, NMD3, NME1, NMT1, NOL11, NOL7, NOL8, NOLC1, NONO, NPM1, NUDCD1, NUDCD2, NUDT5, NUP54, NUP62, NUP88, NUP98, NXT1, OPA3, OSBP, PA2G4, PAICS, PAK1IP1, PARK7, PCBP1, PCBP2, PCGF2, PCM1, PDAP1, PDCL3, PDRG1, PELO, PELP1, PET112L, PFAS, PFKFB4, PGLS, PGRMC2, PHF23, PHF5A, PHPT1, PIK3R2, PKN2, PLK4, PMAIP1, PMVK, PNN, POLDIP3, POLE3, POLR2E, POLR2I, POLR3E, POLRMT, POP7, PPIB, PPIH, PPIL1, PPP2R5E, PPRC1, PRCC, PSMA1, PSMA3, PSMA4, PSMB1, PSMB2, PSMB5, PSMD7, PSMD8, PSPH, PTGER4, PTPN2, PWP1, QTRTD1, RAB34, RAB3GAP2, RAD23B, RAD52, RANGAP1, RASGRP2, RBM14, RBM3, RCBTB2, RCC1, RECQL, RECQL5, RFXANK, RFXAP, RHEB, RIOK1, RIOK2, RNF40, RNF41, RPL10A, RPL18A, RPL22, RPL26L1, RPL29, RPL36, RPL37, RPL37A, RPL5, RPL7L1, RPLP0, RPP14, RPS15, RPS15A, RPS19, RPS21, RPS24, RPS26, RPS6KB1, RPS8, RPUSD4, RSL1D1, RSU1, RTN4, SAC3D1, SAFB, SAP18, SAP30, SART1, SART3, SBDS, SCAMP3, SCAND2, SDCCAG1, SDHB, SEC63, SF1, SF3B14, SF3B4, SF3B5, SFRS1, SFRS2, SFRS3, SFRS7, SFRS9, SHQ1, SLC29A1, SLC38A5, SNAPC1, SNAPC5, SNRPA1, SNRPB, SNRPC, SNRPE, SON, SPATA5L1, SPTLC1, SSNA1, SSSCA1, STIP1, STK17B, SUGT1, SUPT16H, SUPV3L1, TAF1, TAF9, TARS, TBL1XR1, TCERG1, TCP1, TDG, TFAM, TFB2M, TFG, TIMM44, TIMM8B, TIMM9, TMEM69, TNPO2, TNPO3, TOMM70A, TPR, TRAF7, TRAPPC4, TRAT1, TRIM28, TSSC4, TTC21B, TWISTNB, TYSND1, U2AF2, UBA2, UBAP2, UBE2I, UBQLN4, UCHL3, UCHL5, UQCRC2, UROS, USP36, UTP14A, UXS1, VAPA, VPS35, WARS2, WBP4, WBSCR22, WDR4, WDR46, WDR73, WDR75, XPO6, XRCC5, YARS2, YBX1, YIF1A, YIPF2, YKT6, YTHDF1, YWHAG, ZAP70, ZCCHC7, ZMPSTE24, ZNF248, ZNF263, ZNF281, ZNF3, ZNF416, ZNF576, ZNHIT2, ZZZ3 --

NOTCH1 and RELA	156	ABCF2, AGPAT5, AIP, ALG8, AP2S1, APEX1, ARFIP1, ATM, ATP5G2, ATP9B, BCLAF1, CACYBP, CAMK2D, CBX5, CD28, CDC25A, CDC26, CHMP4A, CHURC1, CNOT7, COG8, COPS7A, COX5B, CSTF3, CYCS, DDX19A, DDX21, DDX56, DHPS, EBAG9, EFTUD2, EIF1AY, EIF2B1, EIF4E, EIF5B, ENSA, ERBB2IP, EXOSC5, FAM36A, FANCL, FTSJ3, FXR1, GGA1, GOPC, GORASP2, GTF3C2, HELLS, HNRPDL, HSPA8, IMP4, IPO4, IVNS1ABP, KIAA1919, LAP3, LCK, LOC93622, LRRC41, LSM4, MAP3K7, MBD1, MDM4, MED28, MRPL1, MRPL17, MRPL2, MRPL24, MRPL34, MRPS12, MRPS33, MSH5, MTHFD1, MTMR4, MYCBP, NCLN, NDUFA7, NDUFB8, NDUFS7, NDUFS8, NFKB2, NME1, NOL7, NOL8, NUDCD1, NUDCD2, NUP88, NUP98, NXT1, OPA3, PAICS, PAPOLA, PARK7, PCBP1, PCM1, PDCL3, PNN, POLR2E, POLR2I, POLR3E, POLRMT, PPP1R10, PPP1R11, PSMA2, PSMB1, PSMD8, PTPN2, PWP1, QTRTD1, RAD23B, RAD52, RAP2B, RB1, RBM3, RBX1, RCBTB2, RFXANK, RIOK1, RNF41, RPL10A, RPL22, RPL7L1, RPS15, RPS24, RPS26, SACM1L, SCAMP3, SDCCAG1, SERP1, SFRS3, SFRS7, SLC29A1, SNAPC5, SNRPA, SNRPE, SPTLC2, SRRM2, SSSCA1, TBL1XR1, TBRG4, TFB1M, TIMM44, TIMM8B, TIMM9, TMEM69, TXN2, TYSND1, U2AF2, UCHL3, UQCRC2, USMG5, VPS35, WDR4, WDR73, XPO6, ZNF3, ZNF688, ZNHIT2
RELA and MYC	561	ABCC4, ABCF2, ACN9, ACOX3, ACTB, ADPRH, AFF3, AGA, AGPAT5, ALG8, ANAPC5, ANKFY1, ANKRD10, ANKRD13C, ANKRD37, ANP32B, ANXA6, AP2S1, APEH, APEX1, API5, APOA1BP, APRT, ARAF, ARHGDIB, ARHGEF7, ARIH2, ARL6IP6, ARMC8, ASPH, ATM, ATP11B, ATP2A2, ATP2A3, ATP5F1, ATP5G2, ATP5G3, ATP5H, ATP5J2, ATPIF1, AVEN, B3GALT6, B4GALT7, BAD, BANF1, BAT4, BCL2L1, BCL2L13, BET1L, BLOC1S1, BMP2K, BOP1, BRE, BRWD1, BUB1, C10orf104, C11orf10, C11orf31, C14orf159, C14orf21, C18orf1, C18orf55, C1orf151, C1orf25, C1orf43, C1orf57, C1orf69, C20orf134, C21orf33, C2orf3, C3orf1, C3orf21, C6orf150, C6orf64, C9orf100, C9orf156, C9orf85, CACYBP, CAD, CALR, CBX8, CCT5, CD164, CD2BP2, CD300A, CD59, CD79B, CDC42SE1, CDKL3, CEPT1, CETN3, CFDP1, CLCN7, CLNS1A, CLPP, CLPX, CLUAP1, COG8, COMMD5, COPS7A, COPS7B, COX11, COX15, COX5B, CREB3L4, CSNK1G3, CSNK2B, CSTF2T, CSTF3, CTSC, CTSS, CUEDC2, CUL4A, CXCR4, CYCS, DAD1, DARS, DCPS, DDX19A, DDX21, DDX54, DDX56, DERL1, DGUOK, DHFR, DHFRL1, DHODH, DHPS, DHRS4, DHX30, DNAJA3, DNAJC14, DNAJC16, DNAJC17, DOM3Z, DPH1, DUT, DZIP3, EFTUD2, EIF4A1, EIF4E, EIF4E2, EIF5B, ELAC1, ELMO2, ENSA, ENY2, ERCC8, ETV6, EXOSC2, EXOSC4, EXOSC5, EXOSC7, FABP5, FAH, FAHD1, FAM3C, FAM82B, FANCC, FAS, FBXO22, FBXO31, FBXO4, FBXW2, FKBP1A, FKBP2, FKBP7, FNDC3B, FTSJ3, FXC1, FXR1, GATAD1, GBE1, GCDH, GFM1, GGA1, GHITM, GNAS, GNB1L, GNL1, GNL3, GOPC, GORASP2, GOSR2, GRK6, GSS, GSTK1, GTF2H4, GTF3A, GTF3C2, GTPBP2, H3F3A, H3F3B, HAX1, HCFC1, HEATR1, HELLS, HIRA, HMBS, HMGB1, HNRPDL, HRSP12, HSF1, HSPA8, HSPE1, HTRA2, ID3, IFRD2, IMMP2L, IMP4, INPP5D, IPO4, IPP, ISG20, ITCH, ITGA4, ITGAL, JAK3, KBTBD6, KCTD18, KDELC2, KHK, KIAA0564, KIAA1524, KIAA1704, KIAA1919, KLF6, KPTN, KRTCAP2, LAP3, LARP1, LMBR1, LMLN, LMNB2, LNPEP, LOC93622, LOH12CR1, LRPAP1, LRRC33, LRRC41, LSM4, LY75, M6PR, MAP3K7, MAP3K7IP1, MAPK1, MAT2A, MATR3, MAX, MBD1, MCCC2, MDC1, MDH2, MDM4, MDN1, ME2, MECR, MED28, MED4, METAP2, METTL3, MKKS, MORG1, MPV17, MRE11A, MRPL1, MRPL12, MRPL17, MRPL18, MRPL2, MRPL20, MRPL24, MRPL27, MRPL34, MRPL40, MRPL43, MRPL52, MRPS12, MRPS14, MRPS15, MRPS21, MRPS22, MRPS26, MRPS33, MRPS7, MSH5, MTBP, MTERFD1, MTERFD2, MTHFD1, MTHFD2L, MTR, MTRF1, MTX1, MYCBP, MYO1G, NAP1L1, NAP1L4, NARG1, NARG2, NBN, NCLN, NDE1, NDUFA7, NDUFAB1, NDUFB1, NDUFB2, NDUFB8, NDUFS7, NDUFS8, NDUFV2, NDUFV3, NGLY1, NME1, NOL10, NOL7, NOL8, NOTCH1, NSD1, NSUN2, NSUN4, NUDCD1, NUDCD2, NUP153, NUP214, NUP88, NUP98, NXT1, OPA3, ORC3L, ORC5L, OTUB1, P4HA1, PAICS, PARK7, PCBP1, PCM1, PCNA, PCNP, PDCL3, PDE6D, PDHB, PEX12, PGM2, PHB, PHF14, PIGF, PIGL, PIGS, PIP5K1A, PMM2, PMS1, PNN, PNPO, POFUT1, POLE4, POLR1B, POLR2C, POLR2E, POLR2I, POLR2K, POLR3B, POLR3E, POLRMT, POP4, PPCS, PPIL6, PPM1A, PRKRIP1, PRMT7, PRPF18, PRPF31, PRR3, PSD4, PSMB1, PSMB4, PSMB8, PSMC5, PSMD4, PSMD8, PSMD9, PSME3, PTDSS1, PTMA, PTPN2, PTPRE, PURB, PUSL1, PWP1, QTRTD1, RAB40C, RABGAP1L, RAD23B, RAD50, RAD52, RAN, RBBP4, RBKS, RBL2, RBM3, RBM33, RCBTB2, RCC2, RCL1, RCN2, RCSD1, RDBP, RFXANK, RG9MTD1, RIF1, RING1, RIOK1, RNF113A, RNF135, RNF41, RPL10A, RPL19, RPL22, RPL23A, RPL31, RPL38, RPL7L1, RPLP2, RPP38, RPS15, RPS24, RPS26, RPS27L, RPS4X, RPUSD2, RQCD1,

		RUNX1, RUVBL2, SCAMP1, SCAMP3, SCFD1, SDCCAG1, SDF4, SDHD, SELT, SEPT7, SF3A2, SF3B3, SFRS3, SFRS7, SFT2D3, SIL1, SIP1, SLC25A13, SLC29A1, SLC30A6, SLC35A2, SLC5A6, SLC9A8, SMEK2, SMPD2, SMYD5, SNAPC5, SNRPD2, SNRPE, SNX3, SNX5, SOD1, SP110, SP3, SPCS2, SPG7, SPN, SRP72, SRRM1, SSSCA1, ST3GAL3, ST7L, STK35, STOML2, STXBP4, SUB1, TAF10, TALDO1, TAP2, TAPBPL, TARBP2, TBL1XR1, TCF3, THUMPD3, TIMM10, TIMM13, TIMM44, TIMM50, TIMM8B, TIMM9, TINF2, TLE3, TMED5, TMED9, TMEM14B, TMEM41A, TMEM43, TMEM69, TMPO, TOMM22, TOR1A, TOR3A, TP53, TPCN1, TPP2, TRAP1, TRIM44, TRNT1, TRUB1, TSN, TUBGCP6, TXN, TXNDC9, TXNL4A, TXNRD1, TYSND1, U2AF2, UBE2D4, UBE2G2, UBE2L6, UBOX5, UCHL3, UCK2, UCRC, UMPS, UQCRC2, UROD, USP4, USP7, VARS, VPS28, VPS35, VTI1A, WARS, WDR1, WDR12, WDR18, WDR33, WDR4, WDR41, WDR73, WDR74, WTAP, WWOX, XPO6, ZFP36L2, ZFYVE26, ZNF226, ZNF230, ZNF234, ZNF259, ZNF3, ZNF302, ZNF346, ZNF397, ZNF41, ZNF561, ZNF584, ZNHIT2, ZRANB3
		--
MYC, NOTCH1, and RELA	117	ABCF2, AGPAT5, ALG8, AP2S1, APEX1, ATM, ATP5G2, CACYBP, COG8, COPS7A, COX5B, CSTF3, CYCS, DDX19A, DDX21, DDX56, DHPS, EFTUD2, EIF4E, EIF5B, ENSA, EXOSC5, FTSJ3, FXR1, GGA1, GOPC, GORASP2, GTF3C2, HELLS, HNRPDL, HSPA8, IMP4, IPO4, KIAA1919, LAP3, LOC93622, LRRC41, LSM4, MAP3K7, MBD1, MDM4, MED28, MRPL1, MRPL17, MRPL2, MRPL24, MRPL34, MRPS12, MRPS33, MSH5, MTHFD1, MYCBP, NCLN, NDUFA7, NDUFB8, NDUFS7, NDUFS8, NME1, NOL7, NOL8, NUDCD1, NUDCD2, NUP88, NUP98, NXT1, OPA3, PAICS, PARK7, PCBP1, PCM1, PDCL3, PNN, POLR2E, POLR2I, POLR3E, POLRMT, PSMB1, PSMD8, PTPN2, PWP1, QTRTD1, RAD23B, RAD52, RBM3, RCBTB2, RFXANK, RIOK1, RNF41, RPL10A, RPL22, RPL7L1, RPS15, RPS24, RPS26, SCAMP3, SDCCAG1, SFRS3, SFRS7, SLC29A1, SNAPC5, SNRPE, SSSCA1, TBL1XR1, TIMM44, TIMM8B, TIMM9, TMEM69, TYSND1, U2AF2, UCHL3, UQCRC2, VPS35, WDR4, WDR73, XPO6, ZNF3, ZNHIT2

Note: In order to obtain reliable but comprehensive lists of genes for each transcription factor, we incorporated union of direct targets from only those gold standards, which were obtained by applying a one-sided t-test for the functional gene expression analysis (i.e. for MYC we used union of gold standards # II and IV; for NOTCH1 – II, IV, and VI; for RELA – II and IV).

Figure S1: Comparison of gene sets of transcriptional targets derived from ten different pathway databases by Jaccard index. In case, where Jaccard index of an overlap could not be determined due to comparison of two empty gene lists, we assigned value 0. Cells are colored according to the Jaccard index, from white (Jaccard index equal to 0) to dark-orange (Jaccard index equal to 1). Each sub-figure gives results for a different transcription factor: **(a)** AR, **(b)** BCL6, **(c)** MYC, **(d)** NOTCH1, **(e)** RELA, **(f)** STAT1, **(g)** TP53.

a

	Ingenuity (All)	TransPath	TransFac	BioCarta	KEGG	WikiPathways	CST	GeneSpring (Expression or Promoter binding)	GeneSpring (Expression and Promoter binding)	Pathway Studio	MetaCore	
Number of genes in a database →	*83*	*31*	*8*	*0*	*1*	*1*	*0*	*11*	*1*	*51*	*441*	
Ingenuity (Transcription)	0.2	0.04	0.04	0	0.06	0	0	0	0	0.03	0	*17*
Ingenuity (All)		0.08	0.02	0	0.01	0	0	0.02	0	0.06	0.05	*83*
TransPath			0.11	0	0.03	0	0	0.02	0	0.04	0.03	*31*
TransFac				0	0.13	0	0	0	0	0.04	0.01	*8*
BioCarta					0	0	0	0	0	0	0	*0*
KEGG						0	0	0	0	0	0	*1*
WikiPathways							0	0	0	0	0	*1*
CST								0	0	0	0	*0*
GeneSpring (Expression or Promoter binding)									0.09	0.02	0.01	*11*
GeneSpring (Expression and Promoter binding)										0	0	*1*
Pathway Studio											0.04	*51*

b

	Ingenuity (All)	TransPath	TransFac	BioCarta	KEGG	WikiPathways	CST	GeneSpring (Expression or Promoter binding)	GeneSpring (Expression and Promoter binding)	Pathway Studio	MetaCore	
Number of genes in a database →	*53*	*5*	*2*	*0*	*0*	*0*	*0*	*17*	*4*	*39*	*132*	
Ingenuity (Transcription)	0.15	0.08	0	0	0	0	0	0.09	0	0.07	0.01	*8*
Ingenuity (All)		0.07	0.04	0	0	0	0	0.08	0.04	0.24	0.2	*53*
TransPath			0	0	0	0	0	0.1	0.13	0.1	0.02	*5*
TransFac				0	0	0	0	0.06	0.2	0.05	0.02	*2*
BioCarta					0	0	0	0	0	0	0	*0*
KEGG						0	0	0	0	0	0	*0*
WikiPathways							0	0	0	0	0	*0*
CST								0	0	0	0	*0*
GeneSpring (Expression or Promoter binding)									0.24	0.1	0.03	*17*
GeneSpring (Expression and Promoter binding)										0.08	0.03	*4*
Pathway Studio											0.11	*39*

c

	Ingenuity (All)	TransPath	TransFac	BioCarta	KEGG	WikiPathways	CST	GeneSpring (Expression or Promoter binding)	GeneSpring (Expression and Promoter binding)	Pathway Studio	MetaCore	
Number of genes in a database →	*493*	*29*	*13*	*4*	*14*	*7*	*6*	*26*	*12*	*197*	*1649*	
Ingenuity (Transcription)	0.18	0.11	0.09	0.02	0.05	0.04	0.04	0.02	0	0.21	0.03	*89*
Ingenuity (All)		0.04	0.02	0.01	0.01	0.01	0.01	0.02	0.01	0.16	0.1	*493*
TransPath			0.08	0.03	0.05	0	0.03	0.06	0.05	0.08	0.01	*29*
TransFac				0.06	0.04	0.05	0.12	0.05	0	0.05	0.01	*13*
BioCarta					0	0.1	0	0	0	0.02	0	*4*
KEGG						0.05	0.18	0	0	0.02	0	*14*
WikiPathways							0.08	0	0	0.03	0	*7*
CST								0	0	0.03	0	*6*
GeneSpring (Expression or Promoter binding)									0.46	0.05	0.01	*26*
GeneSpring (Expression and Promoter binding)										0.02	0	*12*
Pathway Studio											0.06	*197*

d

	Ingenuity (All)	TransPath	TransFac	BioCarta	KEGG	WikiPathways	CST	GeneSpring (Expression or Promoter binding)	GeneSpring (Expression and Promoter binding)	Pathway Studio	MetaCore	
Number of genes in a database →	*84*	*5*	*1*	*4*	*3*	*3*	*9*	*15*	*4*	*210*	*133*	
Ingenuity (Transcription)	0.1	0.08	0	0.09	0.1	0.1	0.21	0.05	0.09	0.03	0.01	*8*
Ingenuity (All)		0.02	0	0.02	0.01	0.01	0.03	0.03	0.02	0.14	0.02	*84*
TransPath			0	0	0	0	0.08	0	0	0.02	0.02	*5*
TransFac				0	0	0	0	0.07	0	0	0	*1*
BioCarta					0.17	0.17	0.18	0.06	0.14	0.01	0.01	*4*
KEGG						1	0.2	0.06	0.17	0.01	0.01	*3*
WikiPathways							0.2	0.06	0.17	0.01	0.01	*3*
CST								0.04	0.08	0.03	0.01	*9*
GeneSpring (Expression or Promoter binding)									0.27	0.02	0.01	*15*
GeneSpring (Expression and Promoter binding)										0	0.01	*4*
Pathway Studio											0.06	*210*

e

	Ingenuity (All)	TransPath	TransFac	BioCarta	KEGG	WikiPathways	CST	GeneSpring (Expression or Promoter binding)	GeneSpring (Expression and Promoter binding)	Pathway Studio	MetaCore	
Number of genes in a database →	*196*	*69*	*42*	*141*	*63*	*13*	*7*	*18*	*0*	*134*	*389*	
Ingenuity (Transcription)	0.09	0.09	0.11	0.05	0.09	0.11	0	0	0	0.07	0.03	*18*
Ingenuity (All)		0.17	0.12	0.1	0.11	0.05	0.03	0.04	0	0.23	0.23	*196*
TransPath			0.22	0.11	0.18	0.12	0.04	0.05	0	0.19	0.1	*69*
TransFac				0.08	0.13	0.12	0	0.03	0	0.14	0.07	*42*
BioCarta					0.26	0.08	0.01	0.02	0	0.13	0.07	*141*
KEGG						0.15	0.04	0.05	0	0.15	0.08	*63*
WikiPathways							0	0.07	0	0.07	0.03	*13*
CST								0.09	0	0.02	0.01	*7*
GeneSpring (Expression or Promoter binding)									0	0.07	0.04	*18*
GeneSpring (Expression and Promoter binding)										0	0	*0*
Pathway Studio											0.16	*134*

f

	Ingenuity (All)	TransPath	TransFac	BioCarta	KEGG	WikiPathways	CST	GeneSpring (Expression or Promoter binding)	GeneSpring (Expression and Promoter binding)	Pathway Studio	MetaCore	
Number of genes in a database →	*95*	*67*	*35*	*3*	*33*	*22*	*2*	*19*	*4*	*152*	*285*	
Ingenuity (Transcription)	0.05	0.03	0.05	0	0	0.04	0	0.09	0.13	0.03	0.01	*5*
Ingenuity (All)		0.19	0.16	0.02	0.05	0.04	0.01	0.03	0.02	0.24	0.16	*95*
TransPath			0.52	0.03	0.04	0.02	0.01	0.04	0.01	0.21	0.17	*67*
TransFac				0.03	0.03	0.04	0	0.02	0.03	0.13	0.11	*35*
BioCarta					0.03	0	0	0	0	0.01	0.01	*3*
KEGG						0.04	0	0.02	0.03	0.05	0.02	*33*
WikiPathways							0	0.05	0.08	0.04	0.03	*22*
CST								0	0	0.01	0	*2*
GeneSpring (Expression or Promoter binding)									0.21	0.06	0.05	*19*
GeneSpring (Expression and Promoter binding)										0.02	0.01	*4*
Pathway Studio											0.24	*152*

g

	Ingenuity (All)	TransPath	TransFac	BioCarta	KEGG	WikiPathways	CST	GeneSpring (Expression or Promoter binding)	GeneSpring (Expression and Promoter binding)	Pathway Studio	MetaCore	
Number of genes in a database →	*416*	*151*	*26*	*13*	*42*	*7*	*3*	*21*	*9*	*447*	*913*	
Ingenuity (Transcription)	0.16	0.17	0.15	0.11	0.14	0.04	0.05	0.02	0.01	0.12	0.06	*65*
Ingenuity (All)		0.17	0.05	0.03	0.09	0.01	0.01	0.03	0.02	0.27	0.18	*416*
TransPath			0.1	0.06	0.11	0.02	0.01	0.04	0.03	0.18	0.09	*151*
TransFac				0.18	0.19	0.1	0.07	0	0	0.05	0.03	*26*
BioCarta					0.12	0.18	0.07	0	0	0.03	0.01	*13*
KEGG						0.07	0.07	0.09	0.09	0.07	0.04	*42*
WikiPathways							0.11	0	0	0.01	0	*7*
CST								0	0	0.01	0	*3*
GeneSpring (Expression or Promoter binding)									0.43	0.02	0.01	*21*
GeneSpring (Expression and Promoter binding)										0.01	0.01	*9*
Pathway Studio											0.21	*447*
